# Stammering, Treatment

**Published:** 1874-12

**Authors:** 


Stammering.—The treatment of this defect is now carried on with much success in France by M. Chervin. His method has been the subject of a favorable report to the Academy of Medicine, in which we find a sketch of the system. The training begins by a respiratory practice, in which the patient learns to steady his voice whilst regulating the respiratory rythm.

Then follows the practice of vowels, which, in fact, constitutes the gymnastics of articular phonation. Lastly comes the demonstration of the functions which the tongue and lips have to perform, and of the shape which the mouth should assume in the pronunciation of each letter of the alphabet. This concludes the initiatory practice. Afterwards we have the combination of letters, vowels and consonants in the different and respective positions which they may occupy; and, finally, words and periods, with the intonation and expression which they may require. The whole consists in gymnastically educating the organs of speech; the excellent results being due not so much to actual muscular work as to the precision with which the practice is carried out. The success depends on an effort of the will on the part of the patient to reproduce with the utmost precision a particular movement. The will of the teacher must take the place of the patient's will, as the latter is unable to regulate the movements dictated by it.

M. Chervin justly remarks that stammering is a kind of chorea of the muscles of respiration and phonation. To remedy this he advises slow and measured gymnastic exercises of respiration, this being the first part of the treatment. It is shown above that he combats the unruly movements of the tongue and lips by subjecting these organs to muscular exercise. This method seems thus perfectly rational, and the government have been advised by the Academy of Medicine to give M. Chervin pecuniary support. —*Lancet.*

